# Initial severity of the Positive and Negative Syndrome Scale (PANSS)-30, its main subscales plus the PANSS-6, and the relationship to subsequent improvement and trial dropout: a pooled participant-level analysis of 18 placebo-controlled risperidone and paliperidone trials

**DOI:** 10.1038/s41398-023-02491-6

**Published:** 2023-06-07

**Authors:** Fredrik Hieronymus, Christoph Ulrich Correll, Søren Dinesen Østergaard

**Affiliations:** 1 Department of Clinical Medicine, Aarhus University, Aarhus, Denmark; 2 Institute of Neuroscience and Physiology, University of Gothenburg, Gothenburg, Sweden; 3 The Zucker Hillside Hospital, Department of Psychiatry, Northwell Health, Glen Oaks, NY USA; 4 Donald and Barbara Zucker School of Medicine at Hofstra/Northwell, Department of Psychiatry and Molecular Medicine, Hempstead, NY USA; 5 Charité Universitätsmedizin Berlin, Department of Child and Adolescent Psychiatry, Berlin, Germany; 6 Department of Affective Disorders, Aarhus University Hospital - Psychiatry, Aarhus, Denmark

**Keywords:** Schizophrenia, Predictive markers

## Abstract

Greater initial severity on the 30-item Positive and Negative Syndrome Scale (PANSS-30) correlates positively with antipsychotic-placebo separation and trial dropout, but it is unknown whether these associations are present also on PANSS-derived subscales. We assessed the relationship between initial severity and antipsychotic-placebo separation as measured by PANSS-30 and four PANSS symptom subscales: the positive (PANSS-POS), negative (PANSS-NEG), general (PANSS-GEN) and 6-item (PANSS-6) subscales, using patient-level data from 18 placebo-controlled risperidone and paliperidone trials. Analysis of covariance in the intention-to-treat population (last-observation-carried-forward) was used to assess antipsychotic-placebo separation and trial dropout. Across 6685 participants (90% schizophrenia, 10% schizoaffective disorder), the initial severity-by-treatment interaction was statistically significant for PANSS-30 (beta: −0.155; *p* < 0.001) and all PANSS subscales (beta range: −0.097 to −0.135; *p*-value range: < 0.001 to 0.002). In all cases, antipsychotic-placebo differences increased with initial severity. Judging by the distribution of relative outcomes (percent remaining symptoms), the interaction was partly explained by an increased chance of responding, but also by larger numerical responses in those who did respond, as initial severity increased. Except for PANSS-NEG, high initial severity on all PANSS scales predicted increased trial dropout, although not statistically significantly so for PANSS-6. In summary, we thus replicate previous findings showing greater initial severity to predict larger antipsychotic-placebo separation and extend these results to four PANSS subscales. For PANSS-POS and PANSS-GEN, but not for PANSS-NEG and PANSS-6, we also replicate the association between initial severity and trial dropout. Patients with low initial negative symptom severity were identified as a group of particular interest for further study since their results diverged most from the average both with regard to antipsychotic-placebo separation (low separation measured by PANSS-NEG) and trial dropout (high level).

## Introduction

The 30-item Positive and Negative Syndrome Scale (PANSS-30) is a commonly used scale that measures the severity of symptoms of schizophrenia across multiple domains [1]. The PANSS-30 is typically subdivided into a positive subscale (7 items), a negative subscale (7 items), and a general psychopathology subscale (16 items). The PANSS-6 is a six-item subscale derived from PANSS-30 via item response theory analysis [2], consisting of three items measuring positive symptoms (P1 Delusions, P2 Conceptual disorganization, and P3 Hallucinatory behavior) and three items measuring negative symptoms (N1 Blunted affect, N4 Passive/apathetic social withdrawal, and N6 Lack of spontaneity and flow of conversation). The PANSS-6 has been shown to have comparable sensitivity to change and representation of remission as the PANSS-30 across a number of different schizophrenia populations (acute, chronic, treatment resistant) and antipsychotic compounds [2–5]. Since the PANSS-6 can be administered using much less time compared to PANSS-30 [6–8], it is a promising candidate for use in measurement-based care, e.g., for tracking symptom progression or improvement over time, in clinical practice [9].

One aspect of the main PANSS subscales, as well as of the PANSS-6, that has not been thoroughly evaluated, is the extent to which they show similar features as the PANSS-30 does across the full severity spectrum commonly included in trials of patients with schizophrenia. Specifically, for PANSS-30, it has been demonstrated that initial symptom severity correlates positively with antipsychotic-placebo separation [10] as well as with trial dropout [11]. Notably, such associations do not always translate to derivative subscales, as was recently demonstrated for the Hamilton Depression Rating Scale in patients with major depressive disorder treated with selective serotonin reuptake inhibitors or duloxetine [12, 13]. Whether there exists similar interactions between antipsychotic efficacy and initial symptom severity also on the main PANSS subscales and PANSS-6, is of interest since that influences what effects can be expected to be observed in individual patients when these abbreviated measures are applied in clinical trials or in clinical care.

While, due to regression to the mean, a positive association between initial symptom severity and absolute change scores might be expected for all treatment groups, the same phenomenon would not explain why such an association would be stronger in patients receiving antipsychotics than in patients receiving placebo. There are two likely explanations for why antipsychotic-placebo separation would increase with initial symptom severity. First, it might be that patients with higher initial symptom severity are more likely to experience true drug effects and/or that they are less likely to have substantial placebo effects, possibly because they are more likely genuinely/severely exacerbated patients. Second, a constant fraction of treatment-responsive patients across the severity spectrum could also yield a numerical association between initial severity and antipsychotic-placebo separation if the response in that fraction is proportional to initial severity. Analyses of the relationship between baseline symptom severity using both relative and absolute outcome measures are necessary to disentangle those possibilities from one another.

In this study, we, therefore, assessed the relationship between initial symptom severity and antipsychotic-placebo separation as measured by PANSS-30, its positive (PANSS-POS), negative (PANSS-NEG), and general (PANSS-GEN) subscales, as well as by PANSS-6. In order to gain a better understanding of potential initial severity-by-treatment interactions, we assessed it using both absolute (mean group differences) and relative (fraction of participants displaying different levels of change from baseline) outcome measures. We also assessed the association between initial severity on PANSS-30 and its four subscales, and risk of trial dropout. We hypothesized that relationships between initial symptom severity on PANSS-30 and the studied outcomes [10, 11] would translate to the studied PANSS-30 subscales and to PANSS-6, but that analyses of absolute and relative outcomes, respectively, might differ regarding their implications as to the value of antipsychotic treatment in patients who are less severely ill at baseline.

## Methods

### Data acquisition

Participant-level data for all industry-sponsored, acute-phase, placebo-controlled trials of risperidone and paliperidone in schizophrenia and schizoaffective disorder were requested via the Yale Open Data Access (YODA) website [14]. Data were provided by Johnson & Johnson via YODA for all 19 requested studies. One study (RIS-USA-1/Study 201) did not employ the PANSS-30 and could therefore not be included in this analysis. To verify accuracy of the data, we compared our results to those from study reports provided by YODA and to those available in public reports from the United States Food and Drug Administration [15–20], the European Medicines Agency [21], and ClinicalTrials.gov [22–24].

### Analyses

We first investigated whether mean antipsychotic-placebo separation was related to initial severity across the five outcome measures: PANSS-30, PANSS-POS, PANSS-NEG, PANSS-GEN and PANSS-6. This was done using analysis of covariance (ANCOVA) on the intention to treat (ITT) population and applying last-observation-carried-forward (LOCF) methodology in case of trial dropout. Week 6 was used as endpoint since most studies had an evaluation at that time-point. For studies that did not conduct an evaluation at week 6, the closest available evaluation was used as endpoint with preference given for later evaluations when equidistant (e.g., week 7 was preferred over week 5 if both were available) (Table 1). Models included fixed factors for study and treatment (antipsychotic or placebo). Initial severity on the corresponding outcome parameter was included as a covariate and the interaction between initial severity and treatment was also included in all models. As a sensitivity analysis, we repeated the analyses also including a factor for treatment modality (long-acting injectable (LAI) or oral (PO)), as well as the three-way interaction between treatment and initial severity, and all corresponding two-way interactions. The parameter of interest was the significance level of the three-way interaction between treatment modality, treatment allocation and initial severity, which corresponds to whether the association was statistically significantly different between studies employing different treatment modalities.

**Table 1:** Included studies.

Study	Diagnosis	Endpoint evaluation (week)	PANSS-30 inclusion criteria	Treatment	Number of participants	Age (SD)	Female *n* (%)
*Paliperidone ER*							
R076477-PSZ-3001	Schizophrenia	6	60 to 120	Paliperidone ER 1.5 mg	54	NA^a^	24 (44)
				Paliperidone ER 3 or 6^b^ mg	48	NA^a^	17 (35)
				Paliperidone ER 6 or 12^b^ mg	48	NA^a^	14 (29)
				Placebo	51	NA^a^	28 (55)
R076477-SCA-3001	Schizoaffective disorder	6	> = 60	Paliperidone ER 3–6 mg	108	37.6 (9.5)	37 (34)
				Paliperidone ER 9–12 mg	99	36.5 (10.2)	34 (34)
				Placebo	107	36.3 (10.3)	40 (37)
R076477-SCA-3002	Schizoaffective disorder	6	>= 60	Paliperidone ER 3–12 mg	216	37.3 (8.9)	96 (44)
				Placebo	95	36.1 (10.5)	39 (41)
R076477-SCH-3015	Schizophrenia	6	No PANSS-30 criteria	Paliperidone ER 6–12 mg	160	35.8 (11.7)	53 (33)
				Quetiapine 50–800 mg	159	36.9 (10.3)	50 (31)
				Placebo	79	36.1 (10.5)	29 (37)
R076477-SCH-302	Schizophrenia	6	70 to 120	Paliperidone ER 3–12 mg	76	70.0 (5.0)	56 (74)
				Placebo	38	69.1 (3.4)	27 (71)
R076477-SCH-303	Schizophrenia	6	70 to 120	Olanzapine 10 mg	127	35.8 (11.8)	68 (54)
				Paliperidone ER 6 mg	123	36.8 (10.7)	62 (50)
				Paliperidone ER 9 mg	122	38.0 (11.5)	50 (41)
				Paliperidone ER 12 mg	130	35.5 (10.9)	60 (46)
				Placebo	127	37.3 (11.3)	61 (48)
R076477-SCH-304	Schizophrenia	6	70 to 120	Olanzapine 10 mg	112	40.4 (10.8)	22 (20)
				Paliperidone ER 6 mg	113	42.1 (10.1)	36 (32)
				Paliperidone ER 12 mg	114	41.4 (10.7)	34 (30)
				Placebo	110	42.1 (10.9)	23 (21)
R076477-SCH-305	Schizophrenia	6	70 to 120	Olanzapine 10 mg	128	36.8 (10.3)	31 (24)
				Paliperidone ER 3 mg	127	36.2 (10.9)	46 (36)
				Paliperidone ER 9 mg	125	36.0 (10.9)	44 (35)
				Paliperidone ER 15 mg	114	37.7 (9.8)	41 (36)
				Placebo	123	37.5 (11.2)	37 (30)
R076477-SCH-4012	Schizophrenia	6	70 to 120	Paliperidone ER 1.5 mg	66	41.3 (11.0)	16 (24)
				Paliperidone ER 6 mg	70	40.3 (12.8)	23 (33)
				Placebo	65	36.7 (11.6)	18 (28)
Paliperidone ER: 9 trials	Schizophrenia: 7 trials Schizoaffective disorder: 2 trials	6 weeks: 9 trials		Paliperidone ER: 18 trial arms Olanzapine: 3 trial arms Quetiapine: 1 trial arm Placebo: 9 trial arms	3234	38.0 (12.8)	1216 (38)
*Paliperidone palmitate*							
PALM-JPN-4	Schizophrenia	5	60 to 120	Paliperidone palmitate 75 mg eq.	163	45.7 (13.6)	60 (37)
				Placebo	161	44.1 (12.4)	80 (49)
R092670-PSY-3003	Schizophrenia	7	70 to 120	Paliperidone palmitate 50 mg eq.	94	38.7 (10.5)	28 (30)
				Paliperidone palmitate 100 mg eq.	97	38.7 (10.7)	33 (34)
				Paliperidone palmitate 150 mg eq.	30	41.0 (11.5)	8 (27)
				Placebo	135	40.3 (11.1)	40 (30)
R092670-PSY-3004	Schizophrenia	7	70 to 120	Paliperidone palmitate 25 mg eq.	131	40.3 (10.8)	45 (34)
				Paliperidone palmitate 50 mg eq.	129	38.5 (11.9)	34 (26)
				Paliperidone palmitate 100 mg eq.	130	41.6 (11.0)	46 (35)
				Placebo	126	40.3 (11.8)	48 (38)
R092670-PSY-3007	Schizophrenia	5	70 to 120	Paliperidone palmitate 25 mg eq.	160	38.7 (10.4)	44 (28)
				Paliperidone palmitate 100 mg eq.	165	38.3 (10.5)	55 (33)
				Paliperidone palmitate 150 mg eq.	162	39.0 (11.0)	57 (35)
				Placebo	164	39.3 (11.1)	55 (34)
R092670-SCH-201	Schizophrenia	6	70 to 120	Paliperidone palmitate 50 mg eq.	78	39.0 (10.2)	22 (28)
				Paliperidone palmitate 100 mg eq.	83	37.4 (10.3)	29 (35)
				Placebo	82	39.2 (10.6)	29 (35)
Paliperidone palmitate: 5 trials	Schizophrenia: 5 trials	5 weeks: 2 trials 6 weeks: 1 trial 7 weeks: 2 trials		Paliperidone palmitate: 12 trial arms Placebo: 5 trial arms	2090	40.2 (11.5)	713 (34)
*Risperidone*							
RIS-INT-3	Schizophrenia	6	60 to 120	Haloperidol 20 mg	87	37.6 (9.7)	13 (15)
				Risperidone 2 mg	87	38.4 (10.7)	15 (17)
				Risperidone 6 mg	86	36.9 (10.4)	15 (17)
				Risperidone 10 mg	85	36.3 (9.9)	12 (14)
				Risperidone 16 mg	87	37.3 (10.9)	17 (20)
				Placebo	88	37.2 (10.5)	14 (16)
RIS-SCH-302	Schizophrenia	6	60 to 120	Risperidone 1–3 mg	54	15.7 (1.3)	24 (44)
				Risperidone 4–6 mg	50	15.7 (1.3)	14 (28)
				Placebo	53	15.5 (1.4)	18 (34)
RIS-USA-72	Schizophrenia	4	80 to 120	Risperidone 4 mg	83	38.2 (9.3)	17 (20)
				Risperidone 8 mg	76	37.9 (8.7)	14 (18)
				Placebo	82	38.2 (11.1)	18 (22)
Risperidone: 3 trials	Schizophrenia: 3 trials	4 weeks: 1 trial 6 weeks: 2 trials		Risperidone: 8 trial arms Haloperidol: 1 trial arm Placebo: 3 trial arms	918	33.8 (12.4)	191 (21)
*Risperidone depot*							
RIS-USA-121	Schizophrenia & Schizoaffective disorder	6	60 to 120	Risperidone depot 25 mg	105	39.0 (9.8)	36 (34)
				Risperidone depot 50 mg	118	37.2 (9.4)	27 (23)
				Risperidone depot 75 mg	113	38.5 (10.6)	37 (33)
				Placebo	107	38.1 (9.3)	25 (23)
Risperidone depot: 1 trial	Schizophrenia & Schizoaffective disorder: 1 trial	6 weeks: 1 trial		Risperidone depot: 3 trial arms Placebo: 1 trial arm	443	38.2 (9.8)	125 (28)
Total: 18 trials	Schizophrenia: 15 trials Schizoaffective disorder: 2 trials Schizophrenia & Schizoaffective disorder: 1 trial	4 weeks: 1 trial 5 weeks: 2 trials 6 weeks: 13 trials 7 weeks: 2 trials		Active antipsychotic: 46 trial arms Paliperidone ER: 18 trial arms Paliperidone palmitate: 12 trial arms Risperidone: 8 trial arms Risperidone depot: 3 trial arms Olanzapine: 3 trial arms Quetiapine: 1 trial arm Haloperidol: 1 trial arm Placebo: 18 trial arms	6685	38.1 (12.3)	2245 (34)

*ER* Extended release, *SD* standard deviation. ^a^Individual ages were not included in the data set. Patients were between 12–17 years of age at inclusion. ^b^Dosages varied depending on body weight.

We also partitioned the study population into four groups approximating the four quartiles of the initial severity distribution for each outcome measure. For each such severity group, we calculated the mean antipsychotic-placebo separation in scale points (mean difference, MD) as well as the proportion of participants displaying at least 20% (minor) or at least 50% (major) improvement compared to baseline. Mean antipsychotic-placebo separation was calculated using ANCOVA models that were analogous to those described above, but which did not include initial severity as a covariate. For the two categorical measures, we report pooled unadjusted figures. Since a PANSS item rating of one represents absence of that symptom, percentage based outcome measures were calculated after transforming all PANSS item scores from one to seven to zero to six, so that 100% improvement would correspond to cessation of symptoms.

We also visually assessed relative antipsychotic-placebo separation across all possible relative cut-offs by plotting the cumulative distributions of remaining symptoms (percent) as a function of initial severity quartile and treatment, for each outcome measure. For visualization purposes, data was binned in increments of 5%. Similarly, since worsening is comparatively uncommon but can take on a wide range of percentage values compared to baseline, all instances of deterioration are collapsed into the 105% point on the X-axis. For the same initial severity quartiles, and using the same 5% groupings, we also plotted the difference in the cumulative distributions between treatments.

Finally, we present mean time until trial dropout stratified by treatment and initial severity quartiles for the five outcome measures, and we also regressed time of the last evaluation onto initial severity for each of the five outcome measures using ANCOVA. The latter analyses were adjusted for between-study differences and were stratified by treatment.

Prompted by a negative association between initial PANSS-NEG severity and time to trial dropout for participants receiving antipsychotics, we also conducted 1) a post hoc correlation analysis of baseline scores on the three PANSS subscales (positive, negative and general symptoms) using Pearson’s r, and 2) follow-up analyses of time to trial dropout for the sum-score of the three negative items (PANSS-6-NEG) and the three positive items (PANSS-6-POS), respectively, of the PANSS-6. Prompted by a suggestion from a reviewer, we also repeated the primary analyses using the Marder Negative Symptom Factor (NSF) as an outcome– which is similar to PANSS-NEG, except that it excludes items N05 Difficulty in Abstract Thinking and N07 Stereotyped Thinking and instead includes G07 Motor Retardation and G16 Active Social Avoidance.

Analyses were performed using R version 3.6.3 via remote access to the YODA Data Sharing Environment. *P*-values are two-tailed and due to the strong interdependence of the investigated outcomes (based on the full PANSS-30 and its subscales), we did not adjust for multiple testing.

## Results

### Study population

Details on the eighteen randomized, placebo-controlled trials comprising 6685 participants (90% with schizophrenia and 10% with schizoaffective disorder, mean age: 38.1 ± 12.3 years, 33.6% female) are provided in Table 1. In short, fifteen trials included patients with schizophrenia, two included patients with schizoaffective disorder, and one included patients with either schizophrenia or schizoaffective disorder. Thirteen of the included trials had an evaluation scheduled at week 6, two trials had an evaluation scheduled at week 5 or week 7, respectively, and one trial had its last scheduled evaluation at week 4.

All 18 trials were conducted in exacerbated patients with schizophrenia. Fifteen out of eighteen trials enforced an inclusion criterion of a PANSS-30 score between 60, 70 or 80 to 120, with 70 to 120 being the most common (nine out of eighteen trials). For two trials, a PANSS-30 score of more than 60 was required for inclusion, while for one trial there was no inclusion criterion directly related to the PANSS-30 score. That trial instead applied a CGI-S score of at least 5 as an inclusion criterion. Three trials (R076477-SCA-3001, R076477-SCA-3002 and R076477-SCH-3015) required a score of at least four points on at least two out of P4 Excitement, P7 Hostility, G4 Tension, G8 Uncooperativeness or G14 Poor Impulse Control, while one trial (RIS-USA-72) required a score of at least four points on at least two out of P2 Hallucinatory Behavior, P3 Conceptual Disorganization, G6 Suspiciousness/Persecution or G9 Unusual Thought Content. No trial required that positive symptoms were more prevalent/severe than negative symptoms, or vice versa. Eleven out of eighteen trials specified that the trial only included patients with either an acute exacerbation or acute episode of schizophrenia, while seven trials did not (PALM-JPN-4, R092670-PSY-3003, R092670-PSY-3004, R092670-SCH-201, RIS-INT-3, RIS-USA-72 and RIS-USA-121). These seven trials did, however, enforce the same PANSS-based inclusion criteria and the study report for R092670-PSY-3007, for example, specified that the trial included patients with an acute episode of schizophrenia, defining this as a PANSS-30 score of 70 to 120, whereas studies R092670-PSY-3003 and R092670-PSY-3004 utilized the same PANSS criteria but did not use the term acute episode. Across these 18 trials, 46 active antipsychotic arms were compared with 18 placebo arms. The 46 active antipsychotic trial arms included 18 paliperidone extended release (ER) arms, 12 paliperidone palmitate arms, 8 risperidone arms, 3 risperidone depot arms, 3 olanzapine arms, 1 quetiapine arm, and 1 haloperidol arm.

### Antipsychotic-placebo separation on absolute outcome measures

When looking at mean differences for the full population, there were statistically significant positive associations between initial severity and treatment (i.e., antipsychotic-placebo separation increased with initial severity) for all PANSS measures: PANSS-30 (beta: −0.155 (standard error of the mean (SEM): 0.039); *p* < 0.001), PANSS-6 (beta: -0.135 (SEM: 0.033); *p* < 0.001), PANSS-POS (beta: −0.102 (SEM: 0.032); *p* = 0.002), PANSS-NEG (beta: −0.097 (SEM: 0.026); *p* < 0.001) and PANSS-GEN (beta: −0.125 (SEM: 0.036); *p* < 0.001). In all cases, mean antipsychotic-placebo separation increased with increasing initial severity. Models including treatment modality (LAI or PO) did not find treatment modality to be a statistically significant moderator of the initial severity by treatment interaction (PANSS-30 (*p* = 0.62), PANSS-6 (*p* = 0.20), PANSS-POS (*p* = 0.44), PANSS-NEG (*p* = 0.49), or PANSS-GEN (*p* = 0.40)).

### Antipsychotic-placebo separation on relative outcome measures

Table 2 lists mean antipsychotic-placebo separation as well as the difference between antipsychotic and placebo treated participants regarding the chance of obtaining minor improvement (at least 20% reduction in scale/subscale score) or major improvement (at least 50% reduction in scale/subscale score) compared to baseline, for the four initial severity quartiles. In line with the analyses on absolute outcome measures, mean antipsychotic-placebo separation consistently increased with increasing initial severity. The only exception was PANSS-POS where the second lowest severity group showed larger antipsychotic-placebo separation than the second highest group. Achieving at least minor improvement (> = 20%) was generally more common in the most severe quartile compared to the least severe quartile, with between-quartile differences ranging from −0.2% to 10.6% in favour of the highest severity quartile. The largest between-quartile differences were seen for PANSS-6 (8.3%) and PANSS-NEG (10.6%). Major improvement (> = 50%) also tended to favour the highest severity quartile over the lowest severity quartile, with differences ranging from 2.3% to 6.4%. The largest between quartile differences for major improvement were those for PANSS-30 (5.3%) and PANSS-POS (6.4%).

**Table 2:** Differences in mean antipsychotic-placebo separation and the difference between antipsychotic- and placebo-treated participants regarding the chance of displaying minor improvement (>= 20%) or major improvement (50%).

	Initial severity	Participants^a^	Mean antipsychotic-placebo separation (points)	Antipsychotic-placebo difference in minor improvement (> = 20%)	Antipsychotic-placebo difference in major improvement (> = 50%)
PANSS-30	> = 101	1749	−11.4	21.2%	13.5%
	92 to 100	1709	−8.76	19.9%	8.7%
	83 to 91	1539	−7.76	20.1%	7.4%
	< = 82	1681	−6.74	18.3%	8.2%
PANSS-6	>= 25	1699	−2.77	20.9%	14.8%
	22 to 24	1869	−1.91	18.8%	8.9%
	19 to 21	1695	−1.85	19.7%	8.6%
	< = 18	1417	−1.53	12.6%	10.5%
PANSS-POS	> = 27	1657	−3.52	19.6%	19.9%
	24 to 26	1971	−2.16	11.9%	14.6%
	20 to 23	1419	−3.10	21.9%	15.9%
	< = 19	1632	−2.06	19.8%	13.5%
PANSS-NEG	> = 27	1606	−2.38	20.0%	8.8%
	24 to 26	1826	−1.74	16.1%	5.6%
	20 to 23	1455	−1.86	16.0%	5.3%
	< = 19	1794	−0.91	9.4%	5.6%
PANSS-GEN	> = 51	1808	−5.34	20.8%	11.7%
	46 to 50	1774	−4.04	19.9%	7.1%
	41 to 45	1530	−3.56	16.9%	7.0%
	< = 40	1559	−2.98	16.3%	9.4%

^a^Patient numbers do not add up to 6685 for each severity subgroup due to some items and/or full-scale observations being missing at baseline.

**Fig. 1:**
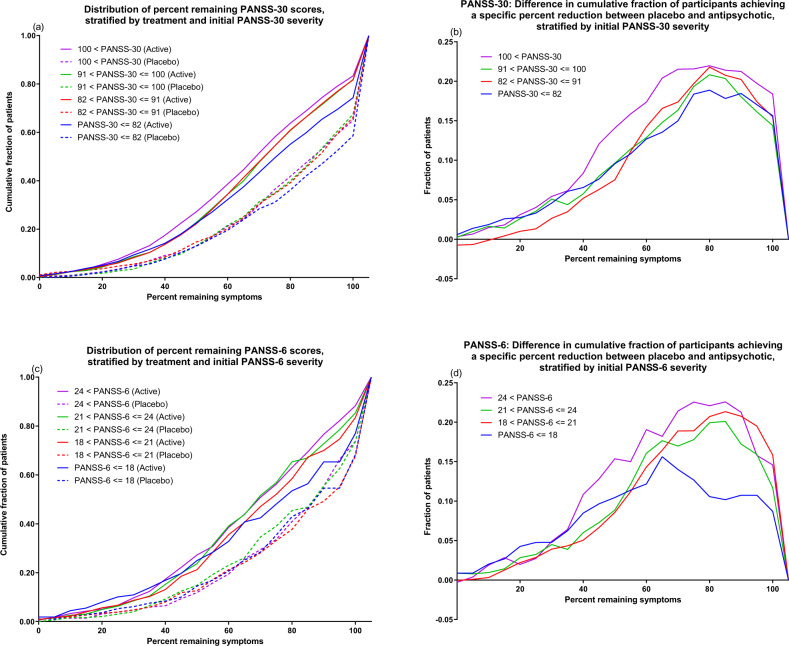
Response distributions for PANSS-30 and PANSS-6. The left-side graphs (**a**, **c**) depict the cumulative distribution of percent remaining symptoms stratified by treatment and initial severity. That is, the height of each line shows how many patients in that group had, e.g., 50% of their baseline symptom severity or lower remaining at endpoint. The right-side graphs (**b**, **d**) show the between-treatment difference in the chance of having achieved at least X percent symptom reduction, i.e., the Y-axis difference between the solid and dotted lines of the same colour. For all graphs, the 105% point on the X-axis contains all instances of worsening as compared to baseline, whether it be a 1% or a 100% increase.

**Fig. 2:**
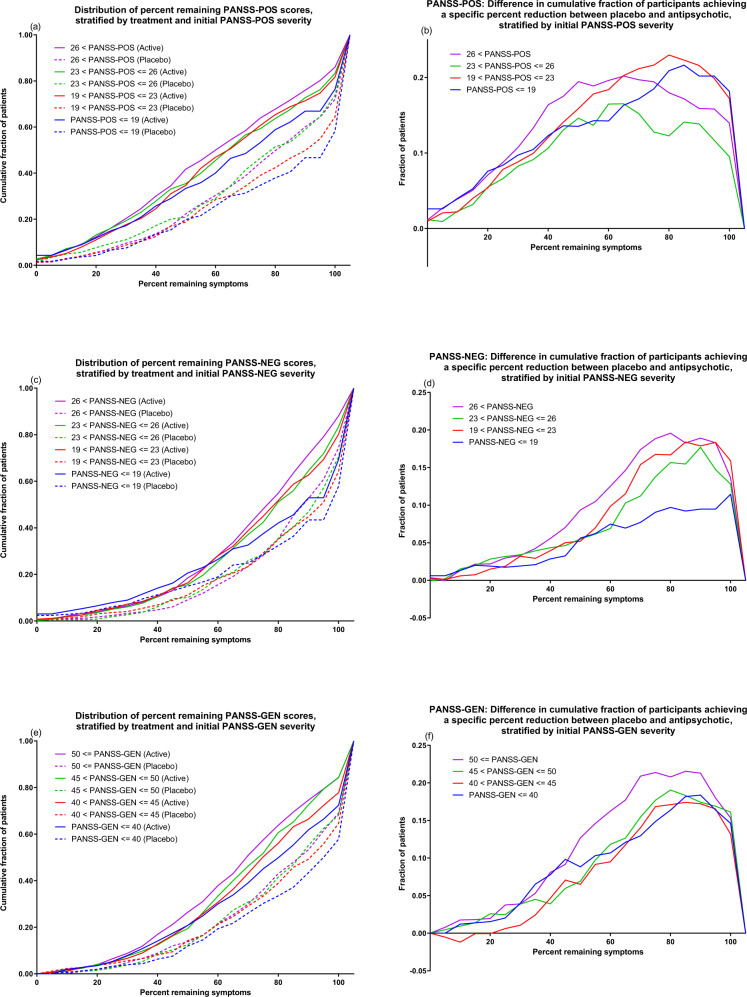
Response distributions for PANSS-POS, PANSS-NEG, and PANSS-GEN. The left-side graphs (**a**, **c**, **e**) depict the cumulative distribution of percent remaining symptoms stratified by treatment and initial severity. That is, the height of each line shows how many patients in that group had, e.g., 50% of their baseline symptom severity or lower remaining at endpoint. The right-side graphs (**b**, **d**, **f**) show the between-treatment difference in the chance of having achieved at least X percent symptom reduction, i.e., the Y-axis difference between the solid and dotted lines of the same colour. For all graphs, the 105% point on the X-axis contains all instances of worsening as compared to baseline, whether it be a 1% or a 100% increase.

Figures 1 & 2 give a visual representation of percent improvement for each initial severity quartile and outcome measure. As a general tendency, participants with higher initial severity (purple and green lines) demonstrated greater percentage improvement compared to participants with lower initial severity (red and blue lines), but this was the case for both those receiving antipsychotic treatment (solid lines) and those receiving placebo (dotted lines). For PANSS-30, PANSS-6 and PANSS-GEN, antipsychotic-placebo separation was most pronounced in the 40% to 80% range of remaining symptoms, as seen by the increasing separation between treatments on the cumulative distributions in that range (Fig. 1a–d; Fig. 2e, f). For PANSS-POS, this pattern was skewed to the left, corresponding to more participants showing large improvements compared to baseline (Fig. 2a, b), while for PANSS-NEG the pattern was skewed to the right (Fig. 2c, d), corresponding to more participants showing small improvements compared to baseline. For all outcome measures and severity quartiles, a substantially larger fraction (approximately 10–15 percentage points) of the participants receiving placebo displayed symptomatic worsening. These observations are grouped in the 105% percent remaining symptoms categories in the figures. In line with results displayed in Table 2, the lowest severity quartile displayed markedly lower antipsychotic-placebo separation for PANSS-6 (Fig. 1d) and PANSS-NEG (Fig. 2d). This was especially pronounced for patients experiencing a low degree of improvement (60–90% remaining symptoms).

### Time to trial dropout

The results of the analyses relating initial severity to time to trial dropout are shown in Table 3. With three exceptions, higher initial severity was statistically significantly associated with shorter time to trial dropout. This was true for both antipsychotic and placebo treatment, although the effect was numerically stronger for placebo-treated participants. The three exceptions were 1) that initial severity on PANSS-NEG predicted statistically significantly longer time to trial dropout for participants receiving antipsychotics, 2) that there was no statistically significant association between initial severity on PANSS-NEG and time to trial dropout for participants receiving placebo, and 3) that there was no statistically significant association between initial PANSS-6 severity and time to trial dropout for participants receiving antipsychotic treatment.

**Table 3:** Time to trial dropout as a function of initial severity.

Severity measure	Mean time in study (weeks) by severity quartile				beta (SEM)	*p*-value
	1^a^	2^b^	3^c^	4^d^		
Active treatment						
PANSS-30	4.75	4.76	4.67	4.66	−0.007 (0.002)	<0.001
PANSS-6	4.69	4.75	4.73	4.66	−0.003 (0.007)	0.665
PANSS-POS	4.92	4.78	4.56	4.54	−0.034 (0.006)	<0.001
PANSS-NEG	4.62	4.66	4.74	4.81	0.010 (0.005)	0.044
PANSS-GEN	4.77	4.74	4.66	4.66	−0.013 (0.004)	<0.001
Placebo						
PANSS-30	4.50	4.39	4.16	4.00	−0.016 (0.004)	<0.001
PANSS-6	4.46	4.32	4.25	3.99	−0.028 (0.012)	0.021
PANSS-POS	4.65	4.22	4.31	3.88	−0.060 (0.010)	<0.001
PANSS-NEG	4.30	4.28	4.29	4.24	0.004 (0.009)	0.675
PANSS-GEN	4.51	4.28	4.31	3.96	−0.026 (0.007)	<0.001

*SEM* Standard error of the mean.

Severity measured by the Positive and Negative Syndrome Scale (PANSS) stratified by PANSS scale and subscale: ^a^PANSS-30 < = 82, PANSS-6 < = 18, PANSS-POS < = 19, PANSS-NEG < = 19, PANSS-GEN < = 40; ^b^PANSS-30 83 to 91, PANSS-6 19 to 21, PANSS-POS 20 to 23, PANSS-NEG 20 to 23, PANSS-GEN 41 to 45; ^c^PANSS-30 92 to 100, PANSS-6 22 to 24, PANSS-POS 24 to 26, PANSS-NEG 24 to 26, PANSS-GEN 46 to 50; ^d^PANSS-30 > = 101, PANSS-6 > = 25, PANSS-POS > = 27, PANSS-NEG > = 27, PANSS-GEN > = 51.

Post hoc analyses found that while baseline scores on PANSS-GEN correlated with both PANSS-POS (*r* = 0.49, 0.47 to 0.51; *p* < 0.001) and PANSS-NEG (*r* = 0.41, 0.39 to 0.43; *p* < 0.001) baseline scores, there was no material or statistically significant correlation between PANSS-POS and PANSS-NEG (*r* = 0.01, −0.01 to 0.03; *p* = 0.401) at baseline. Results from the follow-up analyses of positive (PANSS-6-POS) and negative (PANSS-6-NEG) symptom severity on the PANSS-6 corresponded to the results for PANSS-POS and PANSS-NEG. That is, initial severity on the three positive items of the PANSS-6 was statistically significantly associated with shorter time to trial dropout for both placebo-treated (beta = −0.087 (SEM: 0.018), *p* < 0.001) and antipsychotic-treated (beta = −0.040 (SEM: 0.010), *p* < 0.001) patients. Conversely, initial severity on the three negative items of the PANSS-6 was not statistically significantly associated with time to trial dropout for placebo-treated patients (beta = 0.023 (SEM: 0.017) *p* = 0.179) but was statistically significantly associated with a longer time to dropout for antipsychotic-treated patients (beta = 0.028 (SEM: 0.009), *p* = 0.003). Results from analyses using the Marder NSF were similar to those using PANSS-NEG. The interaction between treatment and initial symptom severity was statistically significant also for the Marder NSF (beta = −0.086 (SEM: 0.025), *p* < 0.001), and Marder NSF severity was positively associated with time to trial dropout for those receiving antipsychotics (beta = 0.011 (SEM: 0.005), *p* = 0.025) but not for those receiving placebo (beta = 0.009 (SEM: 0.009), *p* = 0.276).

## Discussion

In this large-scale participant-level analysis of trials among patients with schizophrenia or schizoaffective disorder, we replicate previous findings of an increased average antipsychotic-placebo separation with increasing initial symptom severity [10] and demonstrate that this holds true also for four subscales of the PANSS-30: PANSS-POS, PANSS-NEG, PANSS-GEN and PANSS-6. With regard to absolute differences, the magnitude of the interaction between initial symptom severity and treatment was similar between PANSS-30 and PANSS-6. Thus, as shown in Table 2, absolute differences in improvement, between the lowest and the highest severity quartile increased by 69% for PANSS-30 (lowest quartile: mean difference 6.7 points; highest quartile: mean difference 11.4 points) and by 81% for PANSS-6 (lowest quartile: mean difference 1.5 points; highest quartile: mean difference 2.8 points).

The most severely ill quartile of participants for each outcome measure also tended to display the largest antipsychotic-placebo separation with respect to both minor (> = 20%) and major (> = 50%) improvement. This tendency was evident also when looking at the distributions of all possible relative outcomes, where the most severely ill quartile generally showed the greatest antipsychotic-placebo separation over relevant parts of the improvement spectrum (Figs. 1 and 2). The differences in relative outcomes were, however, more modest than those seen on absolute outcomes. As an example, while mean improvement relative to placebo on PANSS-GEN increased by 79% from the lowest severity quartile to the highest (Table 2), the difference in the proportion of participants showing minor improvement compared to placebo increased by 28%, and for major improvement the increase was 24%. That mean differences increase more than response rate differences as initial severity increases is expected. Indeed, even if response rates were identical, mean differences would be expected to increase with initial severity since two patients who improve by the same relative magnitude, e.g. by 50%, will both show numerical improvements that are proportional to their own initial severity [25].

Taken together, these findings suggest that initial severity may have some utility in predicting who will respond to treatment. However, except for the lowest severity quartiles for PANSS-NEG and PANSS-6 – which showed decidedly lower antipsychotic-placebo separation than the other three quartiles – differences were not particularly pronounced. Nevertheless, the associations seen in this study are promising and warrant further investigation, but compared to the predictive value from change scores obtained early on in treatment [5, 26, 27], the relative importance of initial severity for predicting subsequent improvement is likely to be low. Future studies assessing severity by treatment interactions in larger datasets may wish to focus specifically on the extremes of the severity distributions, as it seems that this is where there is most variance to be explained. The precision with which these instruments capture disease severity in less symptomatic patients may also be of interest to investigate further using, e.g., item-response theory methodology [28, 29].

The comparatively low rate of improvement for the quartiles of participants with least severe symptomatology according to PANSS-6 and PANSS-NEG is notable. Looking at relative improvement (Figs. 1d and 2d), it seems as if the major difference compared to the other severity quartiles is a shortage of participants with low but positive (60% to 90% remaining symptoms) rates of improvement, and a corresponding increase in the fraction of participants who do not experience any change in symptom severity or who have worsened compared to baseline. This is somewhat unexpected considering that previous studies have found patients with prominent negative symptoms to display less antipsychotic-placebo separation on PANSS-30 than patients with prominent positive symptoms, prominent negative and positive symptoms, or, indeed, patients with no prominent symptoms [30].

The previously observed association between higher initial PANSS-30 severity and increased trial dropout [11] was seen also in this study and the association was stronger in placebo-treated participants (Table 3). The positive relationship between greater initial symptom severity and dropout was seen also on the PANSS-6 (although not for participants receiving antipsychotics), the PANSS-POS and the PANSS-GEN. However, PANSS-NEG scores were not statistically significantly associated with time to trial dropout for placebo-treated subjects, whereas contrary to the overall findings, high PANSS-NEG scores predicted longer time to trial dropout in participants receiving antipsychotics. Follow-up analyses subdividing the PANSS-6 into its positive (PANSS-6-POS) and negative component (PANSS-6-NEG) found that the PANSS-6 subscales showed corresponding associations with time to trial dropout as did the positive and negative subscales of the PANSS-30. Relatedly, while initial PANSS-GEN severity correlated strongly with both PANSS-POS and PANSS-NEG scores at baseline, there was no correlation between PANSS-POS and PANSS-NEG at baseline. This lack of correlation has been reported previously for a subset of three of the studies included in this report [31], and is in line with previous findings of low correlation between PANSS items/factors measuring differing aspects of schizophrenia [32, 33].

The inverse relationship between trial dropout and PANSS-NEG, the lack of correlation at baseline between PANSS-POS and PANSS-NEG, as well as the small degree of antipsychotic-placebo separation on negative symptoms in subjects with low initial PANSS-NEG severity is interesting in light of the controversy regarding the possibility of treatment effect heterogeneity for antipsychotics [34, 35]. While this issue was not directly addressed in this study, increased trial retention for patients receiving antipsychotics (but not placebo) as a function of higher initial negative symptom severity, partially uncorrelated symptom profiles at baseline, as well as small direct treatment effects on negative symptoms in patients with low initial negative symptom severity all seem to indicate that there may be inter-individual differences in response to antipsychotics. In the same vein, the comparatively small antipsychotic-placebo separation on negative symptomatology seen in subjects with low initial PANSS-NEG severity (Table 2) may be of relevance when studying differences in efficacy across antipsychotics and symptom domains of schizophrenia [36, 37].

This study has a number of limitations. First, only 5 (11%) of the 46 active antipsychotic arms were of antipsychotics other than risperidone and paliperidone, and the findings may not generalize to other antipsychotics. Second, 15 (33%) of the 46 active antipsychotic arms included LAI paliperidone or risperidone, which may have influenced antipsychotic-placebo separation as LAI antipsychotics have been associated with lower treatment discontinuation than oral antipsychotics [38, 39]. However, a sensitivity analysis including treatment modality (LAI or PO) did not find this factor to be a moderator of the results. Third, many studies employed a PANSS-30 cut-off for inclusion (often 70 to 120 on PANSS-30; see Table 1), which limits generalizability and introduces the possibility that some participants may have been rated too high or too low in order to permit their inclusion [40]. Fourth, four of the eighteen included studies (R076477-SCA-3001, R076477-SCA-3002, R076477-SCH-3015, RIS-SCH-302) enforced a PANSS item-based entry criterion, which may have led to the preferential inclusion of patients with prominent or predominant positive symptoms, and there are also other factors – e.g., that antipsychotics are generally considered to be more efficacious for positive symptoms – which may have favoured the inclusion of patients with prominent or predominant positive symptomatology. The extent to which the current findings will generalize to an unselected population of patients with schizophrenia is therefore unknown. Fifth, given that all studies included patients with exacerbated schizophrenia, it was not possible to examine the relationship between baseline negative symptoms in patients with predominant negative symptoms, which future studies should do. Sixth, there may be differences in the time trajectory of improvement for different symptom domains – secondary negative symptoms, e.g., may not improve until sometime after a substantial improvement in positive symptoms has been achieved – and the extent to which the current findings will generalize to evaluations at later time-points is, hence, unknown. Finally, we only focused on short-term (4–7-weeks) placebo-controlled trials, further studies are needed to explore the extent to which these findings generalize to longer-term studies and to other populations, such as chronically ill outpatients, those with treatment-resistance and patients followed in active controlled trials or naturalistic treatment settings.

In summary, we replicate previous associations [10, 11], showing that high initial PANSS-30 severity predict both larger antipsychotic-placebo separation, as well as shorter time to trial dropout irrespective of treatment assignment. For mean antipsychotic-placebo separation, the severity by treatment interaction behaved similarly for all four subscale measures, namely the PANSS-POS, PANSS-NEG, PANSS-GEN and PANSS-6. However, when looking at relative outcome measures, the support for a general interaction between initial symptom severity and treatment was less clear-cut and the strongest support for such an interaction was observed at the extremes of the severity distributions. Specifically, antipsychotic-placebo separation was least pronounced in those patients with the lowest quartile scores on PANSS-6 and PANSS-NEG. For trial dropout, PANSS-NEG was also an outlier in that higher initial negative symptom severity predicted longer time to dropout for patients treated with antipsychotics, while not being significantly associated with time to dropout for those treated with placebo. Similarly, initial PANSS-6 severity was also not associated with time to trial dropout in antipsychotic-treated patients. This result was related to the larger contribution of negative symptoms on that subscale compared to the PANSS-30. Further studies focussing specifically on the extremes of the severity distributions, and analysing also individual items, are needed to follow up on these results. Also, studies extending these findings to patients treated with antipsychotics other than risperidone and paliperidone, and to chronically ill outpatients, those with treatment-resistance and patients followed in active controlled trials or naturalistic treatment setting, both short- and longer-term, are warranted.

## Data Availability

The data used in this article can be requested from the Yale Open Data Access website [14].
